# Evaluation of Visual Acuity, Postoperative Refractive Error, and Optical Aberrations in Patients With Previous Corneal Surgery and AcrySof IQ Vivity Intraocular Lens Implantation

**DOI:** 10.1155/joph/3368939

**Published:** 2025-10-17

**Authors:** Miguel Srur, Edison Villagran, Christian Segovia, Cristian Cartes

**Affiliations:** ^1^Departamento de Oftalmología, Centro de la Visión, Santiago, Chile; ^2^Programa de Doctorado en Salud Ecosistémica, Centro de Investigación de Estudios Avanzados Del Maule, Universidad Católica Del Maule, Talca, Chile; ^3^Laboratorio de Microbiología y Parasitología, Departamento de Ciencias Preclínicas, Facultad de Medicina, Universidad Católica Del Maule, Talca, Chile; ^4^Unidad Oftalmología, Departamento de Especialidades, Facultad de Medicina, Universidad de La Frontera, Temuco, Chile

**Keywords:** AcrySof IQ Vivity intraocular lens, cataract surgery, laser-assisted *in situ* keratomileusis

## Abstract

**Purpose:**

To assess the visual outcomes, refractive accuracy, and visual disturbances in patients with a history of myopic laser-assisted in situ keratomileusis (LASIK) who underwent cataract surgery with extended depth of focus intraocular lens (IOL).

**Methods:**

This prospective interventional study included 26 eyes of 13 patients who had previously undergone myopic LASIK surgery. All the participants underwent bilateral phacoemulsification and implantation of the AcrySof IQ Vivity IOL between May 2023 and March 2024. Inclusion criteria included patients > 50 years of age with corneal higher-order aberrations < 0.6 and coma < 0.4. Exclusion criteria included glaucoma, macular disease, retinal detachment, and corneal disease. Visual acuity examinations were performed 1 and 3 months postoperatively.

**Results:**

Before surgery, the mean uncorrected distance visual acuity (UDVA) was 0.3 ± 0.08 logMAR, and the mean corrected distance visual acuity (CDVA) was 0.1 ± 0.03 logMAR. At 3 months follow-up, significant improvements in UDVA (0.04 ± 0.05 logMAR), intermediate (0.1 ± 0.09), and near visual acuity (0.27 ± 0.1) were noted. Postoperatively, 65.4% of the eyes achieved refractive outcomes within ±0.5 D of emmetropia, and 92.3% were within ±1 D. Quality of Vision (QoV) scores revealed no considerable changes following surgery.

**Conclusion:**

The AcrySof IQ Vivity IOL demonstrated good uncorrected distance and intermediate visual outcomes in patients with prior myopic LASIK, along with functionally acceptable near vision and good refractive predictability. These findings support the use of this extended-depth-of-focus lens as a viable solution for presbyopia correction in postrefractive surgery patients, with minimal impact on visual disturbances.

## 1. Introduction

Laser corneal refractive surgery has gained traction for its safety and effectiveness in correcting visual impairments, with millions of patients benefiting from this procedure globally [1]. With an aging patient population, the onset of conditions, such as presbyopia, becomes a substantial concern. Individuals with refractive surgery history are increasingly considering options for correcting presbyopia [2].

Presbyopia correction, especially with techniques such as implanting multifocal intraocular lens (IOL), has emerged as a pivotal area of interest [3]. Despite the benefits of multifocal IOLs in clinical settings for increasing vision at different distances, concerns regarding visual side effects such as decreased contrast sensitivity and the appearance of visual disturbances have prompted a re-evaluation of treatment approaches for this demographic [4, 5]. Moreover, certain contraindications such as glaucoma, retinal disorders, and previous corneal surgeries warrant caution against the straightforward multifocal IOL adoption for correcting presbyopia [6–8].

The use of IOLs for presbyopia correction in patients with previous corneal refractive surgery introduces additional challenges. First, laser-induced corneal alterations may lead to higher-order aberrations, potentially affecting the optical performance of any IOL. The impact of preexisting corneal spherical aberrations should be considered when selecting the IOL design: IOLs with negative spherical aberration may be more suitable after myopic laser-assisted in situ keratomileusis (LASIK), whereas IOLs with neutral or positive spherical aberration could be preferable following hyperopic LASIK [9]. Second, the challenge of achieving accurate postoperative refractive findings is exacerbated by the complexities in calculating the power of the corrective lens [10, 11].

Extended-depth-of-focus (EDOF) lenses, such as AcrySof IQ Vivity IOL, present a promising alternative for correcting presbyopia in patients who have undergone previous photorefractive surgery [12]. Unlike traditional multifocal lenses, EDOF IOLs are designed to provide a continuous vision range from near to distant, thereby decreasing the incidence of visual disturbances, such as halos and glare [13]. This makes them especially appealing for individuals who have undergone procedures such as LASIK for myopia correction and are seeking solutions for age-related presbyopia or cataract development [14]. Their optical design and tolerance to minor refractive errors may facilitate better integration with the altered corneal profiles typical of postrefractive eyes [15]. However, the term EDOF remains controversial, as it lacks standardized optical and functional classification criteria. According to the recent evidence-based framework proposed by the ESCRS Functional Vision Working Group, the Vivity IOL would be more accurately described as a “partial range of field—extend” lens, a classification based on its defocus curve performance rather than optical design features alone [16].

This study mainly aimed to assess the visual acuity and refractive outcomes in patients with a history of myopic LASIK, who were subsequently operated on with the AcrySof IQ Vivity IOL. This approach highlights the study's commitment to advancing the understanding of presbyopia management in patients following refractive surgery and offers valuable evidence for the viability of this IOL as a corrective option.

## 2. Methods

This single-center prospective interventional study examined eyes with a prior history of corneal refractive laser surgery for myopia that underwent EDOF implantation (AcrySof IQ Vivity IOL Alcon) for cataract from May 2023 to March 2024 at Centro de la Vision (Santiago, Chile), and it was registered with ISRCTN registry (29,845,541). The inclusion criteria were as follows: patients > 50 years, undergoing bilateral phacoemulsification, preoperative corneal higher-order aberrations (HOA RMS) < 0.6 microns (6 mm pupil), and coma < 0.4 microns (6 mm pupil). Patients with poorly controlled diabetes mellitus (HbA1c > 7%) and autoimmune conditions (rheumatoid arthritis, lupus, and Sjögren's syndrome) were excluded from the study. Additionally, patients with a history of ocular trauma, corneal inflammation or edema, macular diseases (i.e., macular degeneration and epiretinal membrane), diabetic retinopathy, prior retinal detachment, optic nerve atrophy, chronic uveitis, glaucoma, aniridia, iris atrophy, or severe dry eye were excluded.

Patient assessment included comprehensive ophthalmic examinations before and after Vivity IOL implantation. All patients were operated on by the same experienced surgeon (MS) under topical anesthesia. Patients were followed up at 1 and 3 months to examine visual outcomes. These assessments included binocular uncorrected distance visual acuity (UDVA) and corrected distance visual acuity (CDVA) for far distance (logMAR scale) and binocular uncorrected visual acuity for intermediate (UIVA) and near distance (UNVA) (66 and 40 cm, respectively) using logMAR scale, manifest refraction with spherical equivalent (SE), slit lamp biomicroscopy, tonometry, dilated indirect fundoscopy and dysphotopsia evaluation using a questionnaire (McAlinden Quality of Vision [QoV] questionnaire). Additionally, preoperative corneal topography (Pentacam, Oculus), aberrometry (iTrace, Tracey Technologies), and optical biometry (IOLMaster 700, Carl Zeiss) were performed. The IOL power was calculated using the American Society of Cataract and Refractive Surgeons IOL power calculator, and Barret True-K [17]. The IOL power was verified using an ocular response analyzer (ORA System, Alcon, Fort Worth, TX, USA). In all cases, the target refraction was emmetropia.

This study was conducted in accordance with the Declaration of Helsinki and approved by the Ethical Committee of Centro de la Vision. All participants provided written informed consent before participation.

## 3. Statistical Analysis

Descriptive statistics were used to summarize patient demographics. The normality of the data distribution was examined using the Shapiro–Wilk test, and parametric and nonparametric tests were used accordingly. Statistical significance was set at a *p* value < 0.05. All data were analyzed using Stata (Version 15.0; StataCorp, College Station, TX, USA).

## 4. Results

This study included 26 eyes of 13 patients. The mean age was 58.5 ± 7 years (range 51–78), with 61.5% being women (*n* = 8). The mean UDVA was 0.3 ± 0.08, and the mean CDVA was 0.1 ± 0.03 before surgery. The mean presurgical SE was −1.64 ± 3.1 D, and the mean higher-order aberrations were 0.12 ± 0.13 microns.

Phacoemulsification with an EDOF IOL implantation (AcrySof IQ Vivity IOL, Alcon Laboratories) was uneventful in all cases, with a mean IOL power of 19.8 ± 3.5 D. The CDVA for distance improved to 0.02 ± 0.04 logMAR and 0 ± 0 logMAR at 1- and 3-month follow-up, respectively.

**Figure 1:**
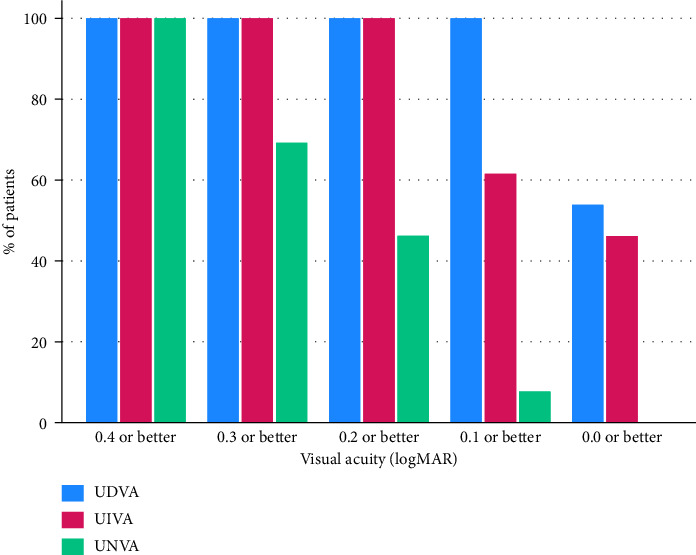
Postoperative binocular uncorrected distance (UDVA), uncorrected intermediate (UIVA), and uncorrected near visual acuity (UNVA) at 3 months follow-up.

The mean postoperative UDVA was 0.03 ± 0.05 logMAR, and the mean postoperative unaided intermediate and near visual acuities were 0.02 ± 0.05 and 0.04 ± 0.05 logMAR, respectively, at 1 month. At 3 months follow-up, the mean postoperative UDVA was 0.04 ± 0.05 logMAR, and the mean postoperative unaided intermediate and near visual acuities were 0.1 ± 0.09 and 0.27 ± 0.1 logMAR, respectively.  Table 1 summarizes the postoperative unaided visual acuities for near, intermediate, and distant vision.  Figure 1 illustrates the distribution of postoperative binocular UDVA, UIVA and UNVA at 3 months follow-up.

**Figure 2:**
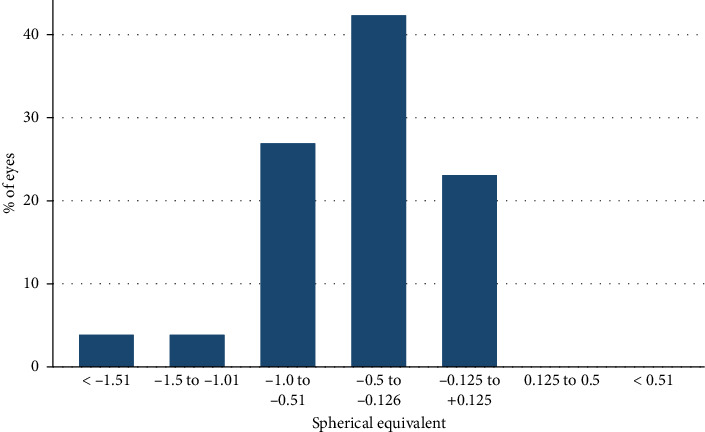
Postoperative spherical equivalent refraction (diopters) at 3-month visit.

The mean SE was −0.46 ± 0.43 D at 3 months postoperatively, demonstrating a significant reduction compared to the preoperative SE (*p*=0.03).  Figure 2 shows the refractive accuracy at 3 months follow-up. Of the eyes studied, 65.4% (*n* = 17) achieved postoperative refraction within ±0.5 D of emmetropia, and 92.3% (*n* = 24) were within ±1 D.

**Figure 3:**
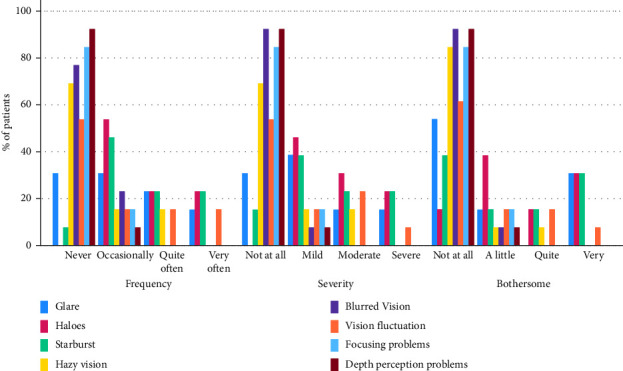
Patients answers to the quality of vision questionnaire at 3-month visit.

The QoV questionnaire score was 21.5 ± 22 before surgery and 19.2 ± 21 at the end of follow-up (*p*=0.18). Occasional haloes (7/13, 53%) and starbursts (6/13, 46%) were the most frequently reported visual disturbances and were mild and slightly bothersome in most patients. Hazy vision, blurry vision, vision fluctuation, focusing problems, and challenges judging distance or depth perception were reported by 30% (4/13), 23% (3/13), 38% (5/13), 15% (2/13), and 7% (1/13) of the patients, respectively (Figure 3). Distortion or double vision was not observed in any patient.

## 5. Discussion

The use of an EDOF IOL following LASIK offers an opportunity to improve intermediate and near visual acuity [15, 18]. However, the reported evidence on visual performance is limited and includes various IOL platforms that are not necessarily comparable. To the best of our knowledge, this is the second study to report the findings of Vivity IOL implantation in patients following myopic LASIK. The former study published by Carreras et al. included 50 eyes of 25 patients and showed good binocular distance and intermediate visual outcomes at 3 months follow-up [19]. These results are consistent with previous findings on this IOL type in eyes without a history of corneal refractive surgery [20–23].

Similar to the results reported by Carreras et al., the postoperative SE in our study was low [19]. Approximately, two-thirds of the eyes achieved postoperative refraction within ±0.5 D, and over 90% were within ±1 D. The minimal residual refractive error postoperatively explained the good UDVA following surgery and the minimal difference in CDVA in our study. Zheng et al. analyzed 41 patients with prior myopic LASIK who underwent EDOF IOL implantation and reported that mild residual refractive errors did not significantly impair visual acuity, highlighting the refractive tolerance of EDOF designs [24]. Although IOL power calculation may be difficult owing to changes in corneal curvature and the anterior–posterior corneal surface ratio, current IOL calculation formulas have demonstrated good predictability in this setting [10, 25, 26]. Additionally, an intraoperative aberrometer may be beneficial for optimizing these calculations by providing real-time refractive measurements during surgery [27].

Regarding intermediate and near vision, the findings were comparable with those of previous reports on eyes with untreated corneas [20–23]. In previously treated patients with myopic LASIK, Carrera et al. reported a mean −0.02 ± 0.09 logMAR at 66 cm, which is consistent with our findings [19]. Regarding near visual acuity, AcrySof IQ Vivity showed commendable performance in patients with virgin corneas. For example, a study by Arrigo et al. demonstrated that patients achieved a mean UNVA of 0.25 ± 0.11 logMAR at 40 cm, enabling the comfortable reading of standard print sizes [21]. Additionally, van Amelsfort et al. noted that using a mini-monovision approach with the Vivity IOL led to a mean UNVA of 0.23 ± 0.12 logMAR, further enhancing near vision capabilities [28]. In patients with previous LASIK, Carrera et al. reported a mean UNVA of 0.27 ± 0.18. This behavior aligns with previous optical simulations, demonstrating an extended range of foci induced by IOL in myopic ablations [12]. Interestingly, improved uncorrected outcomes have been related to residual myopia in the nondominant eye [29, 30]. In our series, residual refractive myopic error may have contributed to the improvement of the uncorrected intermediate and near-vision outcomes through micro-monovision, with a mean residual SE of −0.46. This approach requires further investigation in future studies to optimize outcomes for patients seeking independence from spectacle use.

Patient-reported photic phenomena were relatively mild and infrequent, which is consistent with other studies using similar IOL designs in virgin corneas. Similar to our findings, Carreras et al. reported that visual disturbances, such as starbursts, halos, glare, hazy vision, blurry vision, and double vision, were experienced by 24%, 24%, 28%, 28%, 8%, and 4% of patients with previous myopic LASIK, respectively [19]. Despite the occurrence of these minor disturbances, overall satisfaction is high, with approximately 90% of patients expressing a willingness to undergo the procedure again with the same IOL.

This study had certain limitations. First, the sample size was relatively small, and further research with larger cohorts is necessary to validate these results. Additionally, this was not a comparative study, and including a control group with either no prior refractive surgery or alternative EDOF IOLs may have provided a more robust basis for assessing the specific benefits of the investigated IOL. Furthermore, eyes with significant post-LASIK corneal irregularities were excluded, which restricts the generalizability of our findings to such cases.

In conclusion, the Vivity IOL in patients with LASIK-treated myopia undergoing phacoemulsification exhibited good distance and intermediate vision with functional near-vision while minimizing the visual disturbances commonly related to multifocal IOLs. These outcomes underscore its use in decreasing spectacle dependence and improving the quality of life in this population. Future studies should explore its performance in eyes with higher corneal irregularity degrees, in hyperopic LASIK cases, and compare it with other EDOF IOL designs to determine its relative advantages and clinical applicability. The present results are promising for EDOF lens use in patients with a history of myopic LASIK.

## Figures and Tables

**Table 1:** Mean postoperative spherical equivalent and visual acuity results after Vivity intraocular lens implantation.

SE (D) (3 months)	−0.46 ± 0.43
UDVA (1 month)^∗^	0.03 ± 0.05
UIVA (1 month)^∗^	0.02 ± 0.04
UNVA (1 month)^∗^	0.04 ± 0.05
UDVA (3 months)^∗^	0.04 ± 0.05
UIVA (3 months)^∗^	0.1 ± 0.09
UNVA (3 months)^∗^	0.27 ± 0.1

*Note:* D = diopters, UDVA = uncorrected distance visual acuity, UIVA = uncorrected intermediate visual acuity (60 cm), and UNVA = uncorrected near visual acuity (40 cm).

Abbreviations: SE = spherical equivalent and UDVA = uncorrected distance visual acuity.

^∗^Binocular visual acuity logMAR.

## Data Availability

The data that support the findings of this study are available from the corresponding author upon reasonable request.
